# Progression of arterial toursosity syndrome to multiple aneurysms: Role of defining aortic flow and biomechanics

**DOI:** 10.21542/gcsp.2019.8

**Published:** 2019-03-31

**Authors:** Hamood N.Al Kindi, Amr Elsawy, Yehia R Fahmi, Mazen Abou Gamrah, Soha Romeih, Heba Aguib, Magdi H.Yacoub

**Affiliations:** 1 Aswan Heart Center, Aswan, Egypt; 2 Department of Cardiothoracic Surgery, Sultan Qaboos University Hospital, Muscat, Sultanate of Oman; 3 Department of Cardiac Surgery, Royal Brompton and Harefield NHS Trust, London, UK

## Abstract

Arterial tortuosity syndrome (ATS) is a rare aortopathy characterized by multiple areas of tortuosity, stenosis and aneurysms in large and mid-sized arteries. The management of this syndrome is challenging because its complexity and variability in presentation and progression require a thorough understanding of the biological and biomechanical changes that occur in the arterial system. Here we describe, for the first time, the progression of this disease diagnosed in a 3-year old girl and the use of modern imaging modalities including cardiac magnetic resonance (CMR) 4D Flow, 3D modeling, and computational fluid dynamic simulation to characterize the complex aortic flow and its biomechanics. The integration of these modalities with the clinical evaluation will help in our understanding of this disease and provide patient-specific management.

## Introduction

Arterial tortuosity syndrome (ATS) is a rare autosomal recessive connective tissue disease with about 100 patients reported in the literature. It is characterized by tortuosity, stenosis and aneurysmal formation of large and mid-sized arteries^1^. The effect of flow dynamics and mechano-biological features of the aortic wall in the progression of this disease is not clearly defined. Here we describe the role of advanced modern imaging techniques using three-dimensional segmentation of the aorta, combined with 4D flow and computational fluid dynamic simulation (CFD) in evaluating the aortic flow pattern and aortic wall biomechanics in this complex disease.

### Clinical information

Our patient was a 3-year old girl, who was discovered incidentally to have ATS at the age of 5 days, following a workup for systolic murmur. She was full term and delivered by uneventful cesarean section. She is a product of non-consanguineous marriage and there is no family history of connective tissue disease. The patient did not have any dysmorphic features and was feeding and growing well. She was referred to our center at age 40 days and was followed in the outpatient department over the next three years. All pulses were felt with no radiofemoral delay, and there was no significant blood pressure difference between the upper and lower limbs. The rest of the clinical examination was unremarkable.

Echocardiogram was done on her first evaluation and showed dilatation of the ascending aorta and marked tortuosity of aortic arch and descending aorta. There was mild left ventricular hypertrophy and mild aortic regurgitation. The decision was to manage the patient conservatively with blood pressure control using bisoprolol and captopril, and arrange regular visits in the clinic with follow up CT scans.

## Imaging evaluation

### Methods

The patient had serial CT scans at the age of 40 days, 1 year and 3 years. 3D models were created by the segmentation of the of the three CT scan studies done over the three year follow up using Materialise Mimics and 3-Matic, Leuven, Belgium, Version 21.

CMR 4D flow was performed to assess the pattern of aortic flow using a 1.5-T MRI scanner (Siemens Magnetom Aera; Siemens Medical Systems, Erlangen, Germany) and a Siemens Special Sequence Package for 4D Flow acquisition and post-processing^2^. Quantitative analysis, including peak velocity, peak flow, forward volume, reverse volume and net forward volume, were measured at the ascending aorta, proximal arch, isthmus and descending aorta.

The aortic distensibility and aortic stiffness were measured according to standard techniques and compared to normal values reported previously^3^. Qualitative analysis, including using colour-coded streamlines and vector velocity to visualize flow pattern and vortex development through the aorta, was conducted at four phases: peak systole, representing the maximal flow velocity of the aortic valve, late systole at aortic valve closure, early diastole at the first half of the diastolic time, and late diastole at the second half of the diastolic time.

CFD was used to calculate the flow field in the tortuous aorta and the wall shear stress. We used MRI velocimetry for flow mapping and to get the blood velocity at the aortic root and the descending aorta. The CFD simulation was then created and solved using Ansys CFX^®^ with the proper boundary conditions and mesh parameters.

### Results

**Figure 1.**
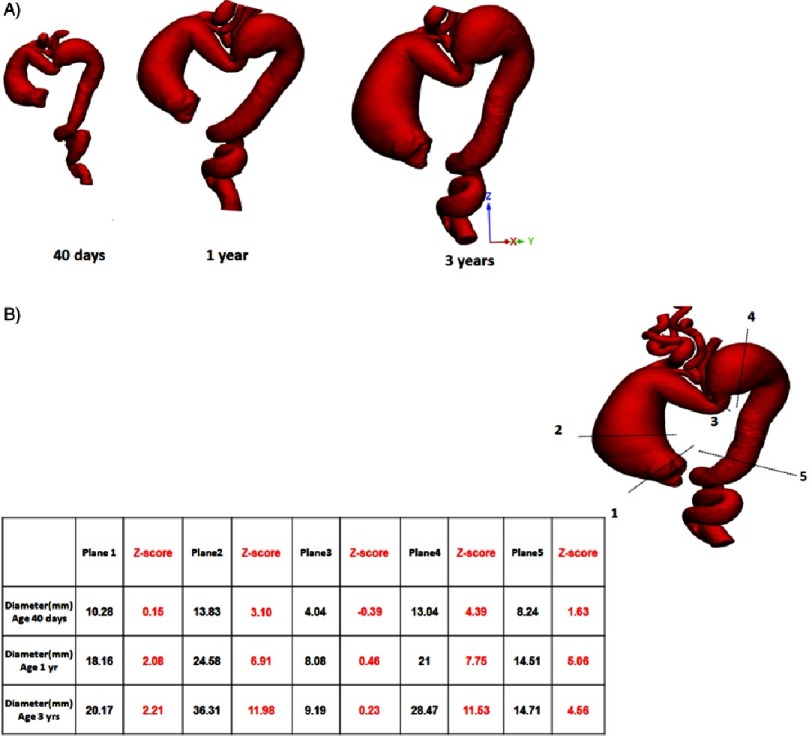
(A) 3-dimensional modelling of the aorta at the age of 40 days, 1 year, and 3 years, showing the progression of the disease and the development of two large aneurysms in the ascending aorta and the proximal descending aorta. (B) The aortic diameters at different planes with the corresponding z scores.

The patient had serial CT scans that showed severe aortic tortuosity with enlargement of both aneurysms in the ascending aorta and proximal descending aorta over the 3-year follow-up. Figures 1A & 1B illustrates the 3D models of the aorta at the age of 40 days, 1 year and 3 years with aortic diameters at different anatomical locations and their Z score values. It illustrates the severe tortuous course and multiple kinks of the aorta and the head vessels. It also illustrates the progression of the disease with the formation of two large aneurysms in the ascending and descending aorta.

**Figure 2.**
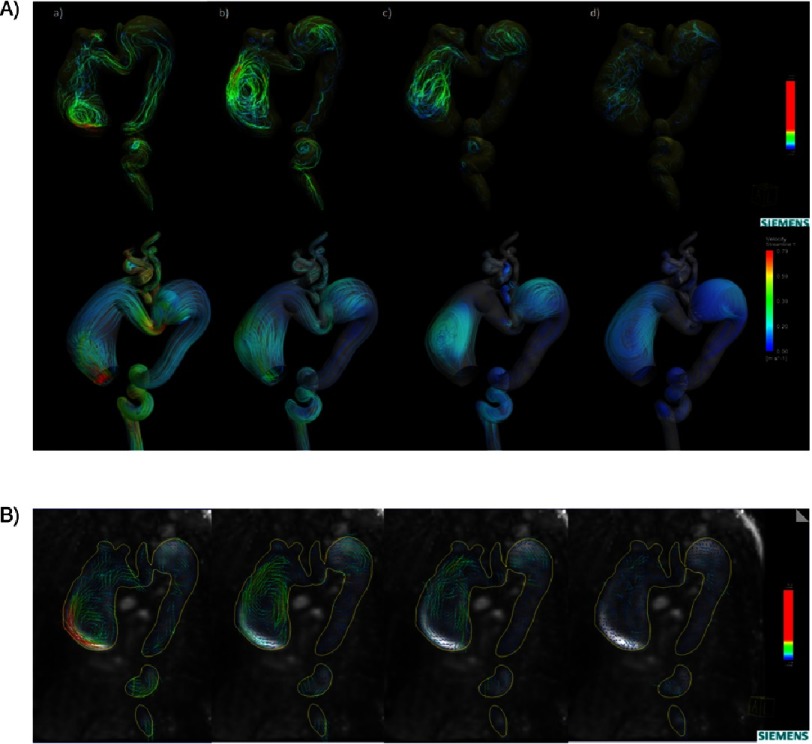
(A) Colour-coded streamlines showing the flow pattern in the ascending aorta, arch and descending aorta during (a) peak systole, (b) late systole, (c) early diastole, and (d) late diastole phases. 4D flow top and CFD bottom (same colour scales). (B) Colour-coded vector velocity showing preoperative flow mapping in the ascending aorta during (a) peak systole, (b) late systole, (c) early diastole, and (d) late diastole phases. A right helical flow with a clockwise rotation of the vortex formed in the dilated ascending aorta and dilated proximal descending aorta starts in the late systole phase and persists throughout diastole.

**Figure 3.**
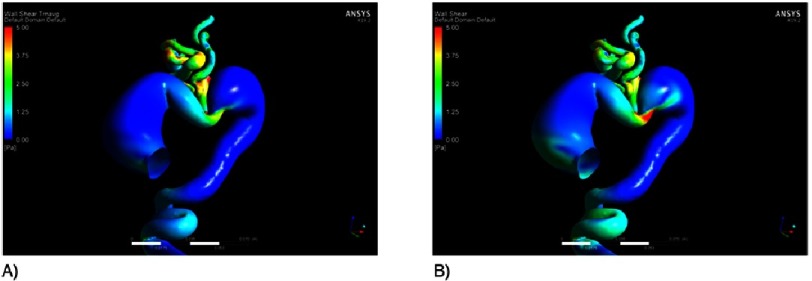
The average (A) and the instantaneous (B) wall shear stress, calculated using CFD showing overall low shear stress in the aorta with highest areas in the aortic isthmus.

In Figure 2, both 4D MRI and CFD showed blood flow velocity aliasing in the proximal ascending aorta early in the cardiac cycle directed to the lateral wall (greater curvature), initiating the large vortex seen in the ascending aorta aneurysm, which was present in 75% of the cardiac cycle. There was another vortex in the proximal descending aorta aneurysm seen in late systole and early diastole. Table 1 summarizes the quantitative analysis of the flow and the aortic distensability at different anatomical planes. The aortic distensibilty was found to be low in different planes of the aorta compared to previously reported age matched normal values^3^. The aortic stiffness, measured using pulse wave velocity (PWV m/s), was high compared to normal age-matched values (9.45 vs 2.88). Figure 3 illustrates the wall shear stress evaluated by CFD that showed overall low wall shear stress in the aorta with highest area in the proximal ascending aorta and the isthmus. The pressure gradient across the area that appeared narrowed in the aortic arch was 8.5 mmHg measured using CFD, which was similar to the blood pressure difference measured clinically between upper and lower limb (10 mmHg).

**Table 1:** Summary of the quantitative analysis of flow and aortic distensability at different anatomical planes.

	Ascending aorta	Proximal arch	Isthmus	Descending aorta
Peak Velocity Magnitude (cm/sec)	80.5	53.5	65.9	36.9
Peak Flow (mL/sec)	90.1	52.0	51.3	40.2
Forward Volume (mL)	18.0	10.0	7.9	7.7
Reverse Volume (mL)	2.5	0.5	1.6	0.0
Net Forward Volume (mL)	15.6	9.4	6.3	7.7
Aortic distensibilty (10^−3^ mmHg ^−1^)	7.4 (normal: 12,04)	2.6 (normal: 9,94)	3.3 (normal: 11,60)	2.1 (normal: 11,14)

**Figure 4.**
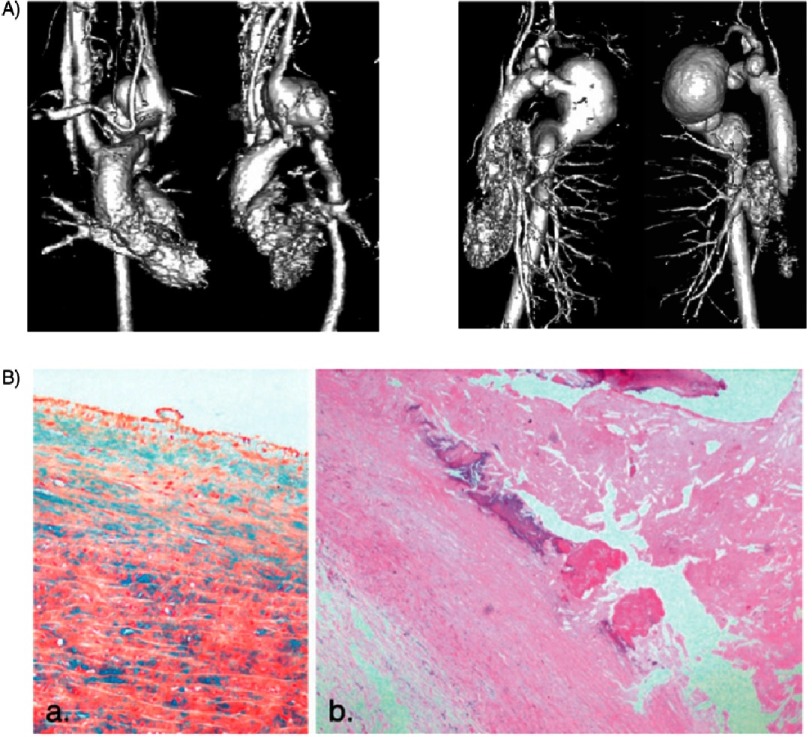
(A) Contrast-enhanced magnetic resonance angiography of the two patients described previously by our group that showed tapering of the aorta in the arch region and then interrupted by multi-lobulated arch aneursyms. (B) Histologic features of the tortuous aortic wall showing the loss of smooth muscle cells and the extensive degeneration and calcification^8^.

### Discussion

Arterial tortuosity syndrome is a very rare autosomal recessive connective tissue disease characterized elongated tortuous large and medium sized arteries with combination of local narrowing and dilated multiple aneurysms^4^. The prognosis and clinical manifestations of ATS are highly variable, ranging from early mortality during infancy to limited manifestations in adulthood. Despite the progressive nature of the aneurysms in this disease, no arterial dissections have been reported in ATS. The main indication for surgical intervention on these patients is the progression of aneurysm dilatation or presence of aortic coarctation^5–7^.

Our group has previously reported the operative outcome of two adult patients, described as having interruption of the aorta with multilobulated arch aneurysms. In retrospect, these patient share the same features of ATS based on their MRI images and histological findings (Figures 4A & 4B)^8^. Histological studies on the aortic tissues showed loss of medial smooth muscle cells, reduction of smooth elastic lamella, and fragmentation of elastic fibers^1,8^.

Unlike other connective tissue disease, including Marfan and Loeys–Dietz, there is an equivocal evidence of the role of TGF-β signaling in ATS^1,9^. Therefore, medical treatment with beta-blockers and ACE inhibitors might not affect the aneurysmal dilatation in this syndrome. Further work on the molecular basis of ATS is needed to add new insights for diagnosis and the pathophysiology of the complex disease.

4D and CFD have a synergistic value in having comprehensive assessment of aortic flow patterns and wall biomechanics in several aortic diseases^10,11^. In 4D, the real blood flow patterns are measured but limited to the observation of the present time of measurement. CFD allows to create simulated models based on several assumptions to measure flow, vortex formation, pressure gradients, wall shear stress .The blood flow pattern seen in our CFD and 4D studies illustrates the direction of flow and the creation of the vortices that are resulted from the marked tourosity of the aorta. The acute angulation in the course of the aorta created multiple areas of high velocity, followed by significant helical and vortical flow in the aneurysmal regions, similar to the pattern seen in aortic coarctation with post stenotic dilatation^11^.

Aortic tortuosity has abnormal hemodynamic effects even in individuals without cardiovascular disease. It creates an asymmetrical flow profile with abnormal wall shear stress patterns and increased aortic stiffness. Aortic stiffness is known to be an independent predictor of progressive aortic dilatation, and this may explain the progression of the disease found in our study^12^. We have also observed in our CFD study the low wall shear stress in most the tortuous aorta including the areas of vortices and recirculation zones. Boyd et al showed that rupture in infra-renal abdominal aortic aneurysms occurred, not at sites of high wall shear stress, but rather at regions of flow recirculation, where low wall shear stress and thrombus deposition predominated^13^. These findings suggest the possibility of the interaction of the flow pattern, wall shear stress, thrombus formation, aortic wall degeneration and eventually rupture.

In conclusion, our observations on the flow pattern and aortic wall biomechanics help in explaining and predicting the location and the progression of the aneurysmal dilatation in aortic tortuosity syndrome. Comprehensive imaging evaluation using CFD and 4D Flow are valuable tools for patient-specific assessment and management of this complex disease.
